# Exploring anesthesiology nurse’ presenteeism in China: cross-sectional study

**DOI:** 10.1186/s12889-024-19476-9

**Published:** 2024-07-26

**Authors:** Ren Jianlan, Yuan Mei, Yang Chunyan, Xie Rendie, Bai Yiping, Liu Li

**Affiliations:** 1 Department of Anesthesiology, the Affiliated Hospital of Southwest Medical University, Luzhou, China; 2 Anesthesiology and Critical Care Medicine Key Laboratory of Luzhou, Luzhou, China; 3 Department of Cardiology, the Affiliated Hospital of Southwest Medical University, Luzhou, China; 4 The Operating Room, the Affiliated Hospital of Southwest Medical University, Luzhou, China

**Keywords:** Anesthesiology nurses, Presenteeism, Work-family conflict, Social support, Occupational commitment, Stress resilience

## Abstract

**Background:**

The department of anesthesiology is the main battlefield for the treatment of acute and critical patients, with high work risk and high work pressure. Due to the particularity of the working environment and nature of work, medical staff have become a group with a high incidence of occupational exhaustion and presenteeism.

**Objective:**

To investigate the current status of presenteeism among anesthesiology nurses in China and to analyze the related influencing factors.

**Methods:**

Three hundred twelve anesthesiology nurses in Sichuan Province were surveyed by means of general data questionnaire, presenteeism scale, work-family conflict scale, perceived social support scale, occupational commitment scale and stress resistance scale from September to November 2023 by convenience sampling method.

**Results:**

The total score of presenteeism was (14.67 ± 3.92), the score of work-family conflict was (45.44 ± 15.90), the score of professional commitment was (87.28 ± 14.30), and the score of perceived social support was (66.04 ± 12.78). The evaluation score of stress resistance was (73.35 ± 11.54). The results of multivariate analysis showed that age, education, mode of employment, position, overtime hours per week, work-family conflict, perceived social support and stress resistance were the factors that affected the presenteeism of anesthesiology nurses, which could explain 44.1% of the total variation. The position ( *β* = 0.296, *P* < 0.001), overtime hours per week (h) ( *β* = 0.271, *P* < 0.001), perceived social support ( *β* = *-*0.279, *P* < 0.001) turned out as the stronger predictors of presenteeism.

**Conclusion:**

The presenteeism of anesthesiology nurses is at a high level and needs to be further improved. Clinical nursing managers should pay attention to the physical and mental health and special needs of anesthesiology nurses. Interventions are made according to the main influencing factors, so as to reduce the incidence of presenteeism and improve the quality and safety of surgery.

## Introduction

In order to promote the high-quality development of nursing in China and improve the health level of the people. On April 29, 2022, the Ministry of Health of China formulated the National Nursing Development Plan (2021–2025). The Plan points out that it is necessary to adhere to high-quality development, take improving the quality and level of nursing services as the core task, and strengthen the construction of the nursing team [1]. The Department of Anesthesiology is the main place of emergency and critical treatment, which needs sufficient human resources, necessary technology and high-precision equipment to monitor and treat patients with critical surgery. Its work is risky and the work pressure is great. Due to long-term and high-load work, medical staff may suffer from physical fatigue and mental breakdown [2, 3]. It has a great impact on the physical and mental health of nurses and is not conducive to the high-quality development of nursing teams.

Presenteeism, also known as impaired health productivity, was first proposed by Professor Copper [4]. A well-known definition of presenteeism of Burton et al. [5] is “ The loss of productivity due to employee health problems who are present but not fully productive.” In principal, presenteeism refers to people who should take leave for rest due to health problems but still stay at work, which is manifested as low work efficiency and reduced work commitment [4, 6].

Relevant studies have shown that due to the particularity of the working environment and nature of work, medical staff have become a group with a high incidence of occupational exhaustion and presenteeism [7–9]. One study found that nurses are four times more likely to have presenteeism than other professions [10]. Recent findings have revealed a high prevalence of attendance problems in nursing work. Specifically, up to 52.6% of US nurses reported that they had difficulty concentrating at work at some point during the past four weeks [11]. In addition, the situation in Swedish hospitals cannot be ignored, where the incidence of attendance problems was 49% for registered nurses and 47% for assistant nurses [12]. In Portugal, the figure is 55% [13]. One healthcare specialty that has reported a recent spike in burnout in the United States is registered nurse anesthetists (CRNAs). While burnout was found to have a negative impact on the health of the organisation (for example, reduced job satisfaction and absenteeism), as well as reduced quality and satisfaction with patient care [14, 15]. But we found no studies on presenteeism for anesthesiology nurses.

Presenteeism is associated with multiple physical and mental health problems in nurses. Several determinants or correlates of attendance have been identified in past studies. The reasons may be work-related (job insecurity, fear of losing income, strict absenteeism policies, overwork, understaffing, overtime, stress experienced) or based on person-related factors (gender, age, occupation, education, health status), etc. [16]. If this problem can not be found in time and properly dealt with, easy to appear anxiety, occupational tension, passive work and other problems.It not only harms the health of nurses, but also affects the safety of patients and the economic benefits of medical institutions [17, 18].

Previous studies have shown that work-family conflict, professional commitment, social support, and resilience are closely related to nurses' work attitudes and behaviors, especially presenteeism [19, 20]. As the two pillars of life, work and family often lead to conflict if they cannot be effectively balanced, which will become an important stressor in nurses' work and make them show negative attitudes, such as presenteeism. At present, it has been proved that presenteeism is not only related to the level of psychological resilience, but also to the interest and emotion of the individual. Nurses with high resilience can cope with work pressure and challenge with positive attitude, and have deep interest and emotion in nursing profession [21]. At the same time, nurses with firm occupational commitment have strong professional identity and higher work engagement. It is worth mentioning that social support is negatively correlated with attendance, in which perceived social support, as a key part, can predict attendance by affecting individual health [22].

At present, studies on presenteeism among medical staff in China mostly focus on emergency nurses [23], obstetrical nurses [5], ICU nurses [7], pediatric nurses [24], etc. There is still a lack of research on the status quo and influencing factors of presenteeism among anesthesiology nurses.The purpose of this study is to known the current situation of presenteeism among nurses in the department of anesthesiology. To provide evidence for improving the quality of anesthesia nursing and ensuring the safety of surgical patients.

## Method

### Study design and participants

The sample size required for estimating the overall rate was then calculated according to the formula. The permissible error was set at 0.06 with* α* = 0.05, calculated *n* = 266, considering the failure rate of 15%, the required sample size is 312.

This was a cross-sectional study. A total of 312 nurses in the department of anesthesiology from 12 3A Grade hospitals (3A Grade hospital is a medical institution level classified in accordance with the current Hospital Grading Management Measures in China. It is the highest level among hospitals in China that are classified into three levels and six grades) in Sichuan Province, including West China Hospital, Sichuan Provincial People’s Hospital, West China Second University Hospital, The Affiliated Hospital of Southwest Medical University, Affiliated Hospital of North Sichuan Medical College, Luzhou People's Hospital, Mianyang Central Hospital, The Affiliated Traditional Chinese Medicine Hospital of Southwest Medical University, Deyang People's Hospital, Dazhou Central Hospital, Sichuan Cancer Hospital, Hospital of Chengdu University of Traditional Chinese Medicine, were selected as the study participants by convenient sampling method from September to November 2023.

Inclusion criteria:

1. Possessing the qualification of nurse practitioner;
2. Working years in the department of anesthesiology ≥ 1 month;
3. Informed consent and voluntary participation in this study.

Exclusion criteria:

1. Studying, practicing and training nurses;
2. Nurses who went out for long-term study or asked for sick leave, maternity leave or personal leave during the investigation period;
3. With serious organic disease, mental or psychological disease in the past.

### Measures

#### General information questionnaire

Based on literature research and expert consultation, the study group designed a general information questionnaire. Including age, gender, number of children, marital status, education, mode of employment, position, years of work, number of night shifts per month, overtime hours per week, satisfaction with the current work situation, and whether they have suffered workplace violence in the past year.

For continuous variables, age was assessed by providing participants with the following three categories: (1) < 30 years; (2) 30-40years; (3) > 40 years. Education was assessed by asking participants about their highest educational level achieved: (1) Secondary and tertiary education; (2) Bachelor's degree; (3) Graduate and above. Number of children was assessed by providing participants with the following three categories: (1) 0; (2) 1; (3) ≥ 2. Years of work was assessed by providing participants with the following three categories:(1) < 5 years; (2) 5–10 years; (3) > 10 years. Night shifts per month was assessed by providing participants with the following two categories: (1) 0–5; (2) 5–10. Overtime hours per week (h) was assessed by providing participants with the following five categories: (1) < 5 h; (2) 5-10 h; (3) 10-15 h; (4) 15-20 h; (4) > 20 h.

#### The presenteeism scale

The presenteeism of anesthesiology nurses was assessed through the Chinese version of presenteeism scale, which was compiled by Koopman C [25] and introduced and translated into Chinese by Zhao Fang [26]. The Cronbach's α coefficient of the Chinese recessive absenteeism scale was 0.806. The presenteeism scale is a single dimension scale with 6 items, including 4 positive items and 2 negative items. The answer options are completely disagree, disagree, unsure, agree, and completely agree. Positive items are scored as 1, 2, 3, 4 and 5 points successively, while items 5 and 6 are scored in the reverse direction, with a total score of 6–30 points. The higher the score, the greater the presenteeism due to health problems. Among which the total score ≥ 15 is classified as high presenteeism, and the total score < 15 is classified as low presenteeism [27].

#### Work-family conflict scale

The work-family conflict of anesthesiology nurses was assessed through the work-family conflict scale, which was translated and revised by Zhang Hemiao, had a Cronbach's α coefficient of 0.96 [28]. The scale contains 3 dimensions (time-based conflict, pressure-based conflict and behavior-based conflict), with 6 items in each dimension and a total of 18 items. Using the Likert 5-level scoring method, answer options were assigned 1–5 points from "strongly disagree" to "strongly agree" on a total scale of 18–90, with higher scores indicating a higher level of work-family conflict.

#### Occupational commitment scale

The occupational identity of anesthesiology nurses was measured through the Chinese version of occupational commitment scale. The Cronbach's α coefficient of the occupational commitment Scale compiled by Blau G [29] and revised by Chinese scholar Pei Yan is 0.919 [30]. The scale contains 5 dimensions (emotional commitment (6 items), normative commitment (5 items), economic cost commitment (4 items), emotional cost commitment (5 items) and opportunity commitment (4 items)), with a total of 24 items. The Likert 5-level scoring method was used, and the answer options were assigned 1–5 points from "strongly disagree" to "strongly agree", among which the last 4 items were scored in reverse, and the total score was 24–120 points. The higher the score, the higher the degree of occupational commitment.

#### Perceived social support scale

The social support of anesthesiology nurses was measured through the Chinese version of perceived social support scale, which was compiled by Dahlem [31] and the Chinese version was translated and revised by Jiang Qianjin [32]. It has a Cronbach's α coefficient of 0.954 and contains 12 items and mainly evaluates three aspects of individual family support (4 items), friend support (4 items) and other support (4 items). Likert 7-level scoring method was used, and the answer options were assigned 1–7 points from "strongly agree" to "strongly agree", with a total score of 12–84 points. The higher the score, the higher the level of social support. A total score of 61–84 would indicate a high level of support, 37–60 indicate moderate level of support, and 12–36 indicate low level of support.

#### Stress resistance scale

The stress resistance of anesthesiology nurses was measured through the stress resistance scale. The Cronbach's α coefficient of the stress resistance scale of medical staff compiled by Zhu Houqiang was 0.907 [33]. The scale contains 18 items in 4 dimensions, including decision coping (6 items), interpersonal connection (4 items), rational thinking (4 items) and flexible adaptation (4 items). Using the Likert 5-level scoring method. The answer options were assigned 1–5 points from "totally inconsistent" to "completely consistent", and the total score was 18–90 points. The higher the score, the higher the level of stress resilience.

### Ethical consideration

Based on the principle of anonymity and informed consent of the participants, data was collected. This study was approved by the Affiliated Hospital of Southwest Medical University (KY2024153).

### Data analysis

SPSS 22.0 statistical software was used for statistical analysis of the data. The counting data was represented by frequency, and the measurement data conforming to normal distribution was represented by (x ± s). For one-way analysis, Chi-square test, two-independent sample t test and Analysis of Variance were used. Pearson correlation analysis and multiple stratification regression analysis were used to explore the influencing factors of presenteeism among anesthesiology nurses. The score of presenteeism of anesthesiology nurses as the dependent variable, and the variables with statistical significance in the univariate analysis. Work-family conflict, occupational commitment, perceived social support, and stress resilience were divided into independent variables for multiple linear regression. Linear regression analyses were applied and by checking the standardized beta coefficients we were able to estimate and compare the individual and independent effects of all predictors. This procedure was also used to test the relationship between exposure and outcome variables and to assess the explained variance (R squared) of the outcome variable. *P* < 0.05 was considered statistically significant.

## Results

The average score of presenteeism of anesthesiology nurses was (14.67 ± 3.92), among which 51.9% (162/312) of nurses were in the high presenteeism group. The comparison of the presenteeism scores of anesthesiology nurses with different characteristics was shown in Table 1.

**Table 1:** Comparison of presenteeism scores among anesthesiology nurses with different characteristics

Item	n(%)	Presenteeism	t/F	High Presenteeism	x^2^
		Score (x ± s)		n(%)	
Gender			2.560^*^		1.165
Male	24(7.7)	16.63 ± 2.45		15(62.5)	
Female	288(92.3)	14.51 ± 3.98		147(51.0)	
Age (yrs)			8.494^**^		0.803
< 30	51(16.3)	14.00 ± 3.29		24(47.1)	
30–40	207(66.3)	15.28 ± 4.07		111(53.6)	
> 40	54(17.3)	13.00 ± 2.30		27(50.0)	
Marital status			5.645^**^		5.989
Unmarried	63(20.2)	14.05 ± 3.93		27(42.9)	
Married	231(74.0)	15.04 ± 3.81		129(55.8)	
Divorced/widowed	18(5.8)	12.17 ± 4.38		6(33.3)	
Number of children			0.683		1.392
0	72(23.1)	14.21 ± 3.71		33(45.8)	
1	156(50.0)	14.77 ± 4.11		84(53.8)	
≥ 2	84(26.9)	14.89 ± 3.75		45(53.6)	
Education			12.187^**^		7.917
Secondary and tertiary education	27(8.7)	15.22 ± 1.65		12(44.4)	
Undergraduate	267(85.6)	14.34 ± 3.86		135(50.6)	
Graduate and above	18(5.8)	18.83 ± 4.84		15(83.3)	
Mode of employment			-2.375^*^		11.691
Contract	228(73.1)	14.36 ± 3.75		105(46.1)	
Establishment	84(26.9)	15.54 ± 4.26		57(67.9)	
Years of work (years)			0.605		0.367
< 5	30(9.6)	14.10 ± 3.95		15(50.0)	
5–10	108(34.6)	14.94 ± 3.25		54(50.0)	
> 10	174(55.8)	14.60 ± 4.29		93(53.4)	
Professional title			2.497		15.561
Nurse	27(8.7)	13.11 ± 4.12		9(33.3)	
Senior Nurse	192(61.5)	14.56 ± 3.89		96(50.0)	
Supervisor Nurse	81(26.0)	15.30 ± 4.07		45(55.6)	
Associate chief nurse and above	12(3.8)	15.75 ± 0.87		12(100.0)	
Position			-6.073^**^		7.550
Clinical Nurse	294(94.2)	14.36 ± 3.65		147(50.0)	
Nursing administrator	18(5.8)	19.83 ± 4.66		15(83.3)	
Night shifts per month (n)			-0.424		2.167
0–5	288(92.3)	14.65 ± 4.03		153(53.1)	
5–10	24(7.7)	15.00 ± 2.23		9(37.5)	
Overtime hours per week (h)			5.156^**^		6.943
< 5	90(28.8)	13.93 ± 4.72		42(46.7)	
5–10	99(31.7)	13.91 ± 3.68		45(45.5)	
10–15	57(18.3)	15.53 ± 2.77		36(63.2)	
15–20	21(6.7)	15.29 ± 4.41		12(57.1)	
> 20	45(14.4)	16.47 ± 2.86		27(60.0)	
Current working status			-4.609^**^		28.775
Satisfied	189(60.6)	13.87 ± 4.18		75(39.7)	
Dissatisfied	123(39.4)	15.90 ± 3.12		87(70.7)	
Experienced workplace violence in the past year			4.129^**^		22.469
Yes	33(10.6)	17.27 ± 2.17		30(90.9)	
No	279(89.4)	14.37 ± 3.97		132(47.3)	

^*^P < *0.05*

^**^P < *0.01*

In Table 2, it showed that the total score of work-family conflict of anesthesiology nurses was (45.44 ± 15.90) points. The total score of occupational commitment was (87.28 ± 14.30). The total score of perceived social support was (66.04 ± 12.78), including 192 (61.5%) at high support level, 117 (37.5%) at medium support level, and 3 (1.0%) at low support level. The total stress resistance score was (73.35 ± 11.54) points. On a bivariate level, presenteeism was positively correlated with education (*r* = 0.130, *P* = 0.022), mode of employment (*r* = 0.134,* P* = 0.018), position(*r* = 0.326, *P* < 0.001), overtime hours per week (h) (*r* = 0.232, *P* < 0.001), current working status (*r* = 0.253, *P* < 0.001) and the total score of work-family conflict (*r* = 0.447, *P* < 0.001). Particularly the negative correlation with gender (*r* = -0.144,* P* = 0.011), experienced workplace violence in the past year (*r* = -0.228, *P* < 0.001) and the total score of occupational commitment (*r* = -0*.*417, *P* < 0.001), perceived social support (*r* = -0.489, *P* < 0.001) and stress resistance evaluation (*r* = *-*0.420, *P* < 0.001). But because such bivariate correlations might only tell half of the story, we conducted a multiple linear regression analysis to inspect the respective variable’s unique associations with presenteeism.

**Table 2:** Correlation of variables

Variable	Score	Presenteeism *(r)*	*P*
Gender (male;female)		-0.144	0.011
Age (< 30;30–40; > 40)		-0.079	0.164
Marital status (Unmarried; married; Divorced/widowed)		-0.010	0.866
Education (Secondary and tertiary education; Bachelor’s degree; Graduate and above)		0.130	0.022
Mode of employment (Contract; Establishment)		0.134	0.018
Position (Clinical nurse; Nursing administrator)		0.326	< 0.001
Overtime hours per week (h) (< 5;5–10;10–15;15–20; > 20)		0.232	< 0.001
Current working status (Satisfied; Dissatisfied)		0.253	< 0.001
Experienced workplace violence in the past year (Yes; No)		-0.228	< 0.001
Total work-family conflict score (18–90)	45.44 ± 15.90	0.447	< 0.001
Total occupational commitment score (24–120)	87.28 ± 14.30	-0.417	< 0.001
Total Social support score (12–84)	66.04 ± 12.78	-0.489	< 0.001
Total stress resistance score (18–90)	73.35 ± 11.54	-0.420	< 0.001

As can be seen in Table 3, the statistical assumptions were sufficiently satisfied (eg, no multicollinearity among independent variables, Durbin-Watson statistic = 2.141). Overall, the estimated regression model significantly and substantially explained the variance in presenteeism (*R*^*2*^ = 0.441,* P* < 0.001).

**Table 3:** Multivariate analysis of presenteeism among anesthesiology nurses (*n* = 312)

Variable	*B*	*SE*	*β*	*t*	*P*	*95%CI*	*VIF*
Constant	17.903	2.752	6.505		< 0.001	12.487 ~ 23.319	
Age (< 30;30–40; > 40)	-1.099	0.409	-0.163	-2.687	0.008	-1.904 ~ -0.294	2.041
Education (Secondary and tertiary education; Bachelor's degree; Graduate and above)	-1.187	0.523	-0.115	-2.268	0.024	-2.217 ~ -0.157	1.425
Mode of employment (Contract; Establishment)	1.396	0.495	0.158	2.820	0.005	0.422–2.370	1.747
Position (Clinical nurse; Nursing administrator)	4.969	0.864	0.296	5.747	< 0.001	3.267–6.670	1.473
Overtime hours per week (h) (< 5;5–10;10–15;15–20; > 20)	0.785	0.149	0.271	5.261	< 0.001	0.491–1.078	1.473
Work-family conflict (score18-90)	0.036	0.015	0.144	2.371	0.018	0.006–0.065	2.064
Perceived social support (score12-84)	-0.086	0.019	-0.279	-4.614	< 0.001	-0.122 ~ -0.049	2.032
Stress resistance (score18-90)	-0.063	0.022	-0.184	-2.897	0.004	-0.105 ~ -0.020	2.254

Durbin-Watson-Statistics = 2.141, *F* = 19.858, *P* < 0.001, *R* = 0.681, *R*^*2*^ = 0.464, the adjusted *R*^*2*^ = 0.441

The results further revealed that position ( *β* = 0.296, *P* < 0.001), overtime hours per week (h) ( *β* = 0.271, *P* < 0.001), perceived social support ( *β* = *-*0.279, *P* < 0.001) turned out as the stronger predictors of presenteeism by far. The analysis showed that nursing administrators are more likely to show presenteeism than clinical nurses ( *β* = 0.296, *P* < 0.001). Further more, the higher perceived social support ( *β* = *-*0.279, *P* < 0.001) and stress resilience ( *β* = *-*0.184, *P* = 0.004), the less presenteeism can be expected. In addition, other predictors, such as Education, mode of employment and work-family conflict were also found to be significant but not particularly strong predictors of presenteeism (see Table 3). Specifically, the higher education, the less frequently anesthesiology nurses displayed presenteeism ( *β* = -0.115, *P* = 0.024). While work-family conflict represents a significant predictor, it does not appear to be a strong one. In general, the higher work-family conflict score, the more likely to show presenteeism( *β* = 0.144, *P* = 0.018). On the other hand, mode of employment seems to be not a significant predictor. The data also showed that older anesthesiology nurses are less likely to show presenteeism than younger ones ( *β* = *-*0.163, *P* = 0.008).

## Discussion

It seemed that presenteeism was quite common among health professionals in the study population. The results of this study showed that the presenteeism of anesthesiology nurses was at a high level, and 51.9% were in a state of high presenteeism. It was basically consistent with relevant research results [7, 23, 34–37] and higher than the research results of Tang Nan on primary medical staff [27]. This indicated that the presenteeism of nurses in 3A Grade hospitals was a prominent problem. Hospitals and clinical nursing managers should pay enough attention to it and focus on it.

Our analyses could basically confirm the currently available data and results of other studies with respect to the work and also person-related factors [37, 38]. The results support the hypothesis that in particular the work-related factors (eg, overtime) play an important role in the occurrence of presenteeism. On the other hand, it was shown that there are two person-related predictors that are of a certain significance: both the position and perceived social support appear to be important predictors.

Our most important findings corroborate those of previous studies that work-related factors influence the occurrence of presenteeism [39, 40]. Overtime and work-family conflict were found to be significant work-related predictors of presenteeism. Similar studies were presented that work-related factors such as increased time pressure and high work load to presenteeism [37, 41]. Regular overtime, especially when there is no agreement between desired and actual working time, increases the probability of presenteeism. Our results showed that nurses aged 30–40 with overtime hours > 20 h per week were more likely to have presenteeism. The reason might be related to the different social roles of the nurses in this age group. In addition to completing their own high-intensity work, they also needed to shoulder the responsibilities and obligations as children and parents. Under multiple pressures and changing environments, they were prone to role conflict, which affected their physical and mental health and thus presented a state of high presenteeism [42]. It suggested that nursing managers should pay more attention to the overtime work of anesthesiology nurses and take effective measures to intervene, such as regularly analyzing the difficulties encountered by nurses in their work regularly and increasing personnel allocation.

Our results suggest that nursing administrators were more likely to show presenteeism than clinical nurses ( *β* = 0.296, *P* < 0.001), which might be related to the special nature of the job. In China, nursing administrators tend to be older and more experienced than clinical nurses. The older an employee, the higher the relative risk of chronic disease [43]. Some studies showed that the worse a employee’s general state of health is, the higher is the level of presenteeism, this effect is particularly noticeable in people with chronic disease [44, 45]. Due to the irreplace ability of management work, the nursing administrators might result in presenteeism if their working status was not good [46].

Previous studies had found that social support was related to nurses' sense of work value, professional identity, negative emotions, etc. Higher social support was conducive to improving nurses' sense of work value and satisfaction [47, 48]. This study found that the lower level of perceived social support, the higher level of presenteeism of nurses. And the presenteeism was negatively correlated with perceived social support (*r* = -0.279, *P* < 0.001). The higher perceived social support, the less presenteeism could be expected. It suggested that clinical nursing managers should pay attention to the physical and mental health and special needs of anesthesiology nurses. Especially focus on nurses with low social support, build a support system integrating hospital, family and social support [49]. And creating a good atmosphere for nurses from the aspects of work environment, family environment and social interpersonal relationship.

Multivariate analysis showed that education and mode of employment were also found to be significant but not particularly strong predictors of presenteeism. A possible reason might be related to the performance reform of Chinese hospitals. In order to be able to better devote and fulfill their duties, hospitals demand equal pay for equal work. Equal pay for equal work means that when an employer engages in the same kind of work for workers with the same technical and labor proficiency, regardless of gender, age, ethnicity, disability, region, etc., as long as it can provide the same amount of labor in different ways, it will receive the same labor remuneration [50]. Therefore, these two predictors did not seem to strongly influence on presenteeism.

### Limitations

This study used a cross-sectional design and therefore could not establish a causal link, which limited the depth of the study to some extent. In addition, since the study data mainly rely on the recall of the participants, this will inevitably be affected by the memory ability of the individuals, which in turn will produce recall bias, which undoubtedly increases the limitation of the study. Moreover, the majority of participants in this study were women and were recruited mainly from tertiary care hospitals, which may limit the generalizability of our findings. Finally, due to limited time and resources, the data were collected in one province in China, so the representativeness of the sample is limited. In the future, a multi-center, large-scale study could be carried out to more comprehensively assess nurse attendance in the department of anesthesiology.

## Conclusions

The presenteeism of anesthesiology nurses is at a high level and needs to be further improved. Clinical nursing managers should pay attention to the physical and mental health and special needs of anesthesiology nurses. And intervene according to the main influencing factors, so as to reduce the incidence of presenteeism and improve the quality and safety of surgery.

## Data Availability

No datasets were generated or analysed during the current study.
